# Ultra-high-resolution inelastic X-ray scattering at high-repetition-rate self-seeded X-ray free-electron lasers

**DOI:** 10.1107/S1600577515024844

**Published:** 2016-02-12

**Authors:** Oleg Chubar, Gianluca Geloni, Vitali Kocharyan, Anders Madsen, Evgeni Saldin, Svitozar Serkez, Yuri Shvyd’ko, John Sutter

**Affiliations:** a National Synchrotron Light Source II, Brookhaven National Laboratory, Upton, NY 11973, USA; b European X-ray Free-Electron Laser, Albert-Einstein-Ring 19, 22761 Hamburg, Germany; c Deutsches Elektronen-Synchrotron, 22761 Hamburg, Germany; d Advanced Photon Source, Argonne National Laboratory, Argonne, IL 60439, USA; e Diamond Light Source Ltd, Didcot OX11 0DE, UK

**Keywords:** inelastic X-ray scattering, X-ray free-electron laser, X-ray optics

## Abstract

Inelastic X-ray scattering (IXS) is an important tool for studies of equilibrium dynamics in condensed matter. A new spectrometer recently proposed for ultra-high-resolution IXS (UHRIX) has achieved 0.6 meV and 0.25 nm^−1^ spectral and momentum-transfer resolutions, respectively. However, further improvements down to 0.1 meV and 0.02 nm^−1^ are required to close the gap in energy–momentum space between high- and low-frequency probes. It is shown that this goal can be achieved by further optimizing the X-ray optics and by increasing the spectral flux of the incident X-ray pulses. UHRIX performs best at energies from 5 to 10 keV, where a combination of self-seeding and undulator tapering at the SASE-2 beamline of the European XFEL promises up to a 100-fold increase in average spectral flux compared with nominal SASE pulses at saturation, or three orders of magnitude more than what is possible with storage-ring-based radiation sources. Wave-optics calculations show that about 7 × 10^12^ photons s^−1^ in a 90 µeV bandwidth can be achieved on the sample. This will provide unique new possibilities for dynamics studies by IXS.

## Abstract 2

This article explores novel opportunities for ultra-high-resolution inelastic X-ray scattering (IXS) at high-repetition-rate self-seeded XFELs. These next-generation light sources are promising a more than three orders of magnitude increase in average spectral flux compared with what is possible with storage-ring-based radiation sources. In combination with the advanced IXS spectrometer described here, this may become a real game-changer for ultra-high-resolution X-ray spectroscopies, and hence for the studies of dynamics in condensed matter systems.

## 1. Introduction   

**Figure 1:**
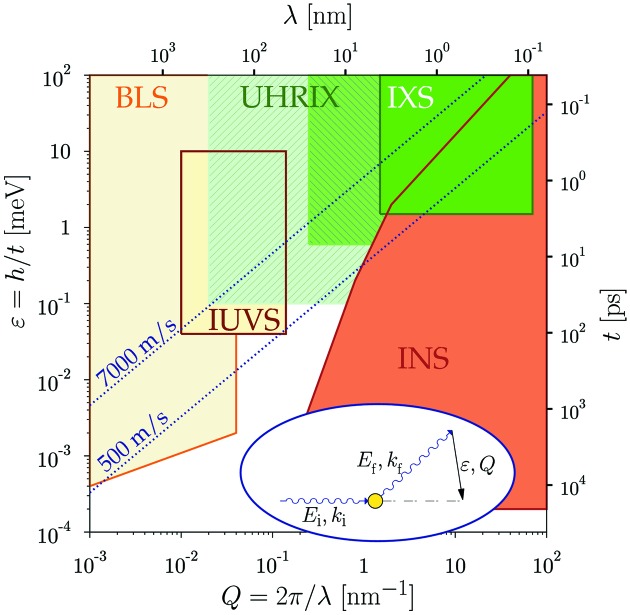
Time–length (*t*–λ) and energy–momentum (∊–*Q*) space of excitations relevant in condensed matter. The figure indicates how different domains are accessed by different inelastic scattering probes: neutrons (INS), X-rays (IXS), ultraviolet (IUVS) and Brillouin light scattering (BLS). The ultra-high-resolution IXS (UHRIX) spectrometer presented by Shvyd’ko *et al.* (2014 ▸) entered the previously inaccessible region marked in shaded green. The novel capabilities discussed in the present paper will enable IXS experiments with 0.1 meV and 0.02 nm^−1^ resolution in the region marked in shaded light green. Hence, they will close the existing gap between the high-frequency and low-frequency probes. The energy ∊ = 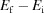 and the momentum 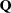 = 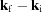 transfers from initial to final photon/neutron states are measured in inelastic scattering experiments, as schematically shown in the inset.

Momentum-resolved inelastic X-ray scattering (IXS) is a technique introduced (Burkel *et al.*, 1987 ▸; Burkel, 1991 ▸) and widely used (Sette *et al.*, 1998 ▸; Burkel, 2000 ▸; Krisch & Sette, 2007 ▸; Monaco, 2015 ▸; Baron, 2015 ▸) at synchrotron radiation facilities for studies of atomic-scale dynamics in condensed matter. IXS is a photon-in/photon-out method applicable to any condensed matter system, whether it is solid, liquid, biological or of any other nature. A photon with energy 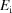 and momentum 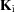 changes its energy and momentum to 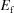 and 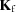 in an inelastic scattering process in the sample and leaves behind a collective excitation with energy 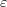 = 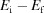 and momentum 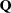 = 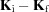, as shown in the sketch in Fig. 1 ▸. The interpretation of IXS is straightforward as it measures the dynamical structure factor 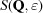, *i.e.* the spatiotemporal Fourier transform of the van Hove time-dependent pair correlation function (Ashcroft & Mermin, 1976 ▸). Therefore, it provides access to dynamics on a length scale 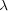 = 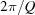 and at a time scale 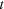 = 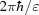.

IXS is one of only a few existing inelastic scattering techniques. Each technique provides access to a limited region in the time–length scale or equivalently in the energy–momentum space of collective excitations relevant for condensed matter. Fig. 1 ▸ shows how a broad range of excitations are covered by different inelastic scattering probes: neutrons (INS), X-rays (IXS), ultraviolet light (IUVS) and Brillouin light scattering (BLS). A gap remains in experimental capabilities between low-frequency (visible and ultraviolet light) and high-frequency (X-rays and neutrons) inelastic scattering techniques. Hence, dynamics in the range from about 1 to 100 ps on atomic- and meso-scales are still inaccessible by any known experimental probe. This is precisely the region of vital importance for disordered systems and therefore many outstanding problems in condensed matter dynamics, such as the nature of the liquid to glass transition, could be addressed by entering this unexplored domain.

In principle there are no limitations preventing IXS from penetrating this unexplored dynamic range of excitations.^1^ This would, however, require solving two longstanding challenges in IXS. First, IXS spectrometers in their traditional implementation rely on an X-ray optics concept utilizing single-bounce Bragg back-reflecting spherical analyzers, leading to pronounced Lorentzian tails of the spectral resolution function. This approach has reached an impasse where the best numbers in energy (∼1.5 meV) and momentum-transfer (∼1.5 nm^−1^) resolutions have not improved for the past 20 years (Masciovecchio *et al.*, 1996 ▸; Said *et al.*, 2011 ▸). Second, the IXS signal is very weak. For example, with ∼10^9^ incident photons there is often less than one photon inelastically scattered into the detector. Hence, more efficient IXS spectrometers with better resolution and more powerful X-ray sources are required to advance the field.

Recently, a new type of dispersive spectrometer was tested. This ultra-high-resolution IXS (UHRIX) spectrometer (Shvyd’ko *et al.*, 2014 ▸) achieved a spectral resolution of 0.6 meV at a momentum transfer down to 0.25 nm^−1^ (shaded green area in Fig. 1 ▸). Additionally, the spectral contrast improved by an order of magnitude compared with traditional IXS spectrometers (Burkel *et al.*, 1987 ▸; Sette *et al.*, 1995 ▸; Masciovecchio *et al.*, 1996 ▸; Baron *et al.*, 2001 ▸; Sinn *et al.*, 2001 ▸; Said *et al.*, 2011 ▸). To sharpen the desired resolution to 0.1 meV and 0.02 nm^−1^ and to ensure higher count rates, we propose to further develop the angular-dispersive X-ray optical scheme (Shvyd’ko *et al.*, 2013 ▸; Stoupin *et al.*, 2013 ▸) replacing scanning IXS spectrometers with broadband imaging spectrographs (Shvyd’ko, 2015 ▸).^2^

In addition to these optics developments, new types of X-ray sources are on the horizon that will overcome the problem of insufficient IXS cross section by delivering a higher spectral flux, namely seeded high-repetition-rate X-ray free-electron lasers (XFELs). Low-gain X-ray free-electron laser oscillators (XFELOs) may in some time in the future produce a spectral flux of up to 10^14^–10^15^ photons s^−1^ meV^−1^ (Kim *et al.*, 2008 ▸; Lindberg *et al.*, 2011 ▸), but currently they are still under conceptual development (Maxwell *et al.*, 2015 ▸). High-gain XFELs, on the other hand, are available today. Self-amplified spontaneous emission (SASE) XFELs (Emma *et al.*, 2010 ▸; Ishikawa *et al.*, 2012 ▸; Altarelli *et al.*, 2006 ▸) deliver light pulses with unprecedented peak power compared with storage-ring-based sources. However, the average photon flux that can be delivered is limited due to the low repetition rate of their linac drivers. By contrast, the European XFEL will adopt superconducting accelerator technology producing 27000 X-ray pulses per second, *i.e.* orders of magnitude above the 120 pulses per second of the LCLS and the 60 pulses per second at SACLA.

The UHRIX instrument with the desired 0.1 meV resolution can be installed at the SASE-2 beamline of the European XFEL together with the MID instrument (Madsen *et al.*, 2013 ▸) operating in the 5–25 keV range. UHRIX performs best at relatively low photon energies between 5 and 10 keV with an optimum around 9 keV. Owing to the high repetition rate of the European XFEL, the nominal average output flux at SASE-2 amounts to about 10^12^ photons s^−1^ meV^−1^ at 9 keV, which is more than one order of magnitude greater than at synchrotron radiation facilities (Baron, 2015 ▸). Furthermore, the spectral flux can be substantially increased by self-seeding (Geloni *et al.*, 2011*a* ▸; Amann *et al.*, 2012 ▸), which at the European XFEL first will be available at the SASE-2 beamline (XFELSEED, 2014 ▸). Another order of magnitude increase in flux is achievable by tapering the magnetic field of the seeded undulator (Sprangle *et al.*, 1979 ▸; Kroll *et al.*, 1981 ▸; Orzechowski *et al.*, 1986 ▸; Fawley *et al.*, 2002 ▸, 2011 ▸; Wang *et al.*, 2009 ▸; Geloni *et al.*, 2010 ▸; Jiao *et al.*, 2012 ▸). We therefore propose an optimized configuration of the SASE-2 X-ray source combining self-seeding and undulator tapering techniques in order to reach more than 10^14^ photons s^−1^ meV^−1^, the same number estimated by Yang & Shvyd’ko (2013 ▸). In combination with the advanced IXS spectrometer described here, this may become a real game-changer for ultra-high-resolution X-ray spectroscopy, for IXS in particular, and hence for the studies of dynamics in dis­ordered systems.

The paper is organized as follows: in §2 we demonstrate that self-seeding, combined with undulator tapering, allows the aforementioned figure of 10^14^ photons per second per meV bandwidth to be achieved at the optimal photon energy range around 9 keV. This result is obtained by numerical modeling using the XFEL code *GENESIS* (Reiche, 1999 ▸) and start-to-end simulations for the European XFEL. In §3 we introduce and evaluate the X-ray optical design to achieve 0.1 meV resolution IXS. The choice of optical elements and their design parameters are studied by dynamical theory calculations for monochromatization in §3.1, and by geometrical optics considerations for X-ray focusing in §3.3. The spectrograph design with a spectral resolution of 0.1 meV in a 5.8 meV-wide spectral window of imaging is presented in §3.4. The design parameters are verified in §3.5 by wavefront propagation simulations from source to sample using a combination of *GENESIS* (Reiche, 1999 ▸) and *SRW* (Chubar & Elleaume, 1998 ▸) codes. All results are summarized and discussed in §4.

## 2. High-average-flux X-ray source for ultra-high-resolution IXS   

### 2.1. Concept   

This section describes a configuration of the SASE-2 X-ray source at the European XFEL, combining hard X-ray self-seeding (HXRSS) and undulator tapering techniques in order to optimize the average output spectral flux around 9 keV, which is the optimum working point of the UHRIX setup. In its simplest configuration a HXRSS setup consists of an input undulator and an output undulator separated by a chicane with a single-crystal monochromator (Geloni *et al.*, 2011*a* ▸). Like this, it has been implemented both at LCLS (Amann *et al.*, 2012 ▸) and at SACLA (Inagaki *et al.*, 2014 ▸). The time structure of the European XFEL is characterized by ten macropulses per second, each macropulse consisting of 2700 pulses, with 4.5 MHz repetition rate inside the macropulse. The energy carried by each pulse and the performance of the crystal cooling system, removing deposited heat between macropulses, should conservatively satisfy the condition that during a macropulse the drift in the central frequency of the crystal transmission function cannot exceed the Darwin width. Then, due to the high repetition rate of the European XFEL, the simplest two-undulator configuration for HXRSS is not optimal and a setup with three undulators separated by two chicanes with monochromators is proposed. This amplification–monochromatization double-cascade scheme is characterized by a small heat load on the crystals and a high spectral purity of the output radiation (Geloni *et al.*, 2011*b* ▸).^3^

The figure of merit to optimize for IXS experiments is the average spectral photon flux. Here, the high repetition rate of the European XFEL yields a clear advantage compared with other XFELs. However, even relying on its high repetition rate, the maximum output of the European XFEL is 10^12^ photons s^−1^ meV^−1^ in SASE mode at saturation, which is too low to satisfy the flux requirements discussed in the previous section. Therefore self-seeding and undulator tapering are needed.

**Figure 2:**
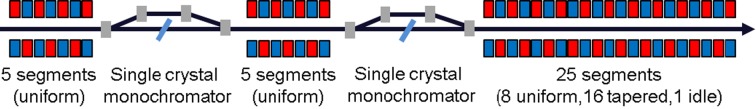
Layout of the SASE-2 undulator (35 segments) in the double-cascade self-seeding scheme for HXRSS. The monochromators are placed in the photon beam in between undulator segments where a magnetic chicane deviates the electrons.

The techniques proposed in this article exploit another unique feature of the European XFEL, namely its very long undulators. The SASE-2 line will feature 35 segments, each consisting of a 5 m-long undulator with 40 mm period. The 175 m SASE-2 undulator is much longer than required to reach saturation at 9 keV (at 17.5 GeV electron energy and 250 pC pulse charge the saturation length amounts to about 60 m). We exploit this additional length to operate the SASE-2 baseline in HXRSS mode followed by post-saturation tapering according to the scheme in Fig. 2 ▸, which has been optimized for our purposes.

As discussed above, since we seek to combine the high repetition rate of the European XFEL with the HXRSS mode of operation, special care must be taken to ensure that the heat load on the crystal does not result in a drift in the central frequency of the transmission function of more than a Darwin width. A preliminary estimate (Sinn, 2012 ▸) showed that in the case of radiation pulses with an energy of a few µJ the heat deposited could be removed by the monochromator cooling system without any problems.^4^ In order to keep the pulse energy impinging on the crystal within the few-µJ range, one can exploit the double-cascade self-seeding setup in Fig. 2 ▸. The setup increases the signal-to-noise ratio, the signal being the seed pulse, competing with the electron beam shot noise. At the position of the second crystal, the seed signal is characterized by a much narrower bandwidth than the competing SASE signal leading to a much higher spectral density. In other words, in the frequency domain, the seed signal level is amplified with respect to the SASE signal by a factor roughly equal to the ratio between the SASE bandwidth and the seed bandwidth. One can take advantage of the increased signal-to-noise figure to reduce the number of segments in the first and second part of the undulator down to five, thus reducing heat load on the crystals due to impinging X-ray pulses. In the simulations we assume that the diamond crystal parameters and the (004) Bragg reflection are similar to those used for self-seeding at LCLS (Amann *et al.*, 2012 ▸). Optimization of crystal thickness and the choice of reflections may yield an increase in the final throughput (Yang & Shvyd’ko, 2013 ▸). However, here we will not be concerned with the optimization of the HXRSS setup in this respect.

### 2.2. Radiation from the SASE-2 undulator   

**Figure 3:**
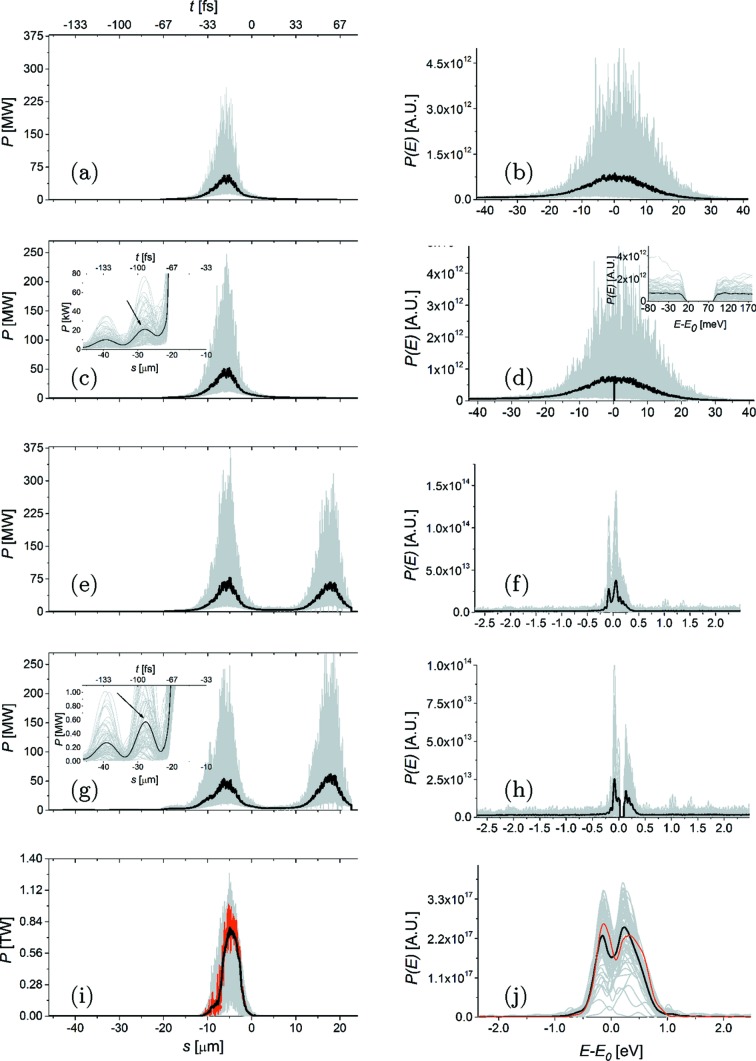
Power distribution and spectrum of the X-ray pulse along the undulator, calculated: (*a*) and (*b*) at the exit of the first undulator (five segments); (*c*) and (*d*) after the first HXRSS monochromator; (*e*) and (*f*) at the exit of the second undulator (five segments); (*g*) and (*h*) after the second HXRSS monochromator; (*i*) and (*j*) at the exit of the setup. Grey lines refer to single-shot realisations, the black line refers to the average over 100 simulations. The insets in (*c*) and (*g*) show an enlarged portion of the main plot, illustrating the seed appearing after the filtering process. The black arrows indicate the position of the seed relative to the electron slice with maximum current. The red lines in graphs (*i*) and (*j*) refer to the particular XFEL pulse that is used for wavefront propagation simulations (see §3).

We performed numerical simulations of the high-average-flux source in Fig. 2 ▸ using the *GENESIS* code (Reiche, 1999 ▸). Simulations are based on a statistical analysis consisting of 100 runs. Start-to-end simulations (Zagorodnov, 2012 ▸) yielded information about the electron beam; see Table 1 ▸ that is used as input for *GENESIS*. The parameters pertaining to the double-cascade self-seeded operation mode studied in this paper are shown in Table 1 ▸. The first five undulator segments serve as a SASE radiator yielding the output power and spectrum shown in Figs. 3(*a*) and 3(*b*) ▸, respectively. As explained in the previous section, when working at high repetition rates it is critical to minimize the energy per pulse impinging on the diamond crystals. The energy per pulse can easily be evaluated integrating the power distribution in Fig. 3(*a*) ▸ yielding an average of about 1.2 µJ per pulse. As discussed in the previous section, this level of energy per pulse is fully consistent with the proposed setup. The filtering process performed by the first crystal is illustrated in Figs. 3(*c*) and 3(*d*) ▸. The X-ray pulse then proceeds through the second undulator as shown in Fig. 2 ▸, where it seeds the electron beam.

Power and spectrum at the exit of the second undulator are shown in Figs. 3(*e*) and 3(*f*) ▸, respectively. This figure illustrates the competition between seed amplification and the SASE process, given the relatively low seeded pulse power from the first part of the setup. This is particularly evident in the time domain, where the seeded pulse follows about 20 µm after the SASE pulse with almost similar power levels. Moreover, each of the pulses (seeded and SASE) carries about the same energy as the initial SASE pulse incident on the first crystal with a total incident average energy per pulse of about 2.7 µJ, *i.e.* still within the heat-load limits discussed in the previous section. In the frequency domain a greatly increased peak power spectral density is observed for the seeded signal [compare Figs. 3(*d*) and 3(*f*) ▸] while the SASE pulse contributes a wide-bandwidth noisy background. The fact that the power spectral density for the seed signal is larger than for SASE by about an order of magnitude (roughly corresponding to the ratio of the SASE bandwidth to the seeded bandwidth) is what actually allows the X-ray beam to impinge on the second HXRSS crystal at low power, but with a large signal-to-noise (seeded-to-SASE) ratio, thus reducing heat loading effects by about one order of magnitude compared with a single-chicane scheme.

The filtering process performed by the second crystal is illustrated in Figs. 3(*g*) and 3(*h*) ▸. After this, the seed signal is amplified to saturation and beyond, exploiting a combination of HXRSS with post-saturation tapering.

**Figure 4:**
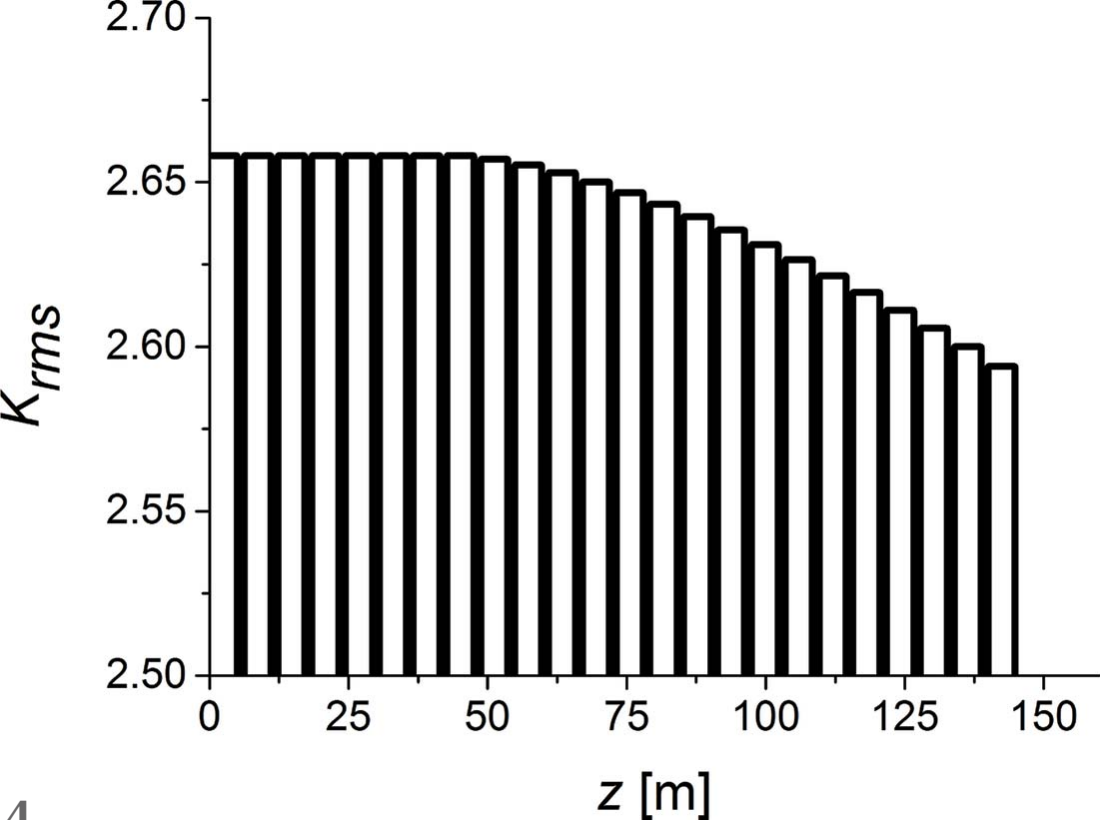
Taper configuration for the output undulator (25 segments: 8 uniform, 16 tapered, 1 idle).

Tapering is implemented by changing the *K* parameter of the undulator, segment by segment according to Fig. 4 ▸. The tapering law used in this work has been implemented on an empirical basis, in order to optimize the spectral density of the output signal. The use of tapering together with monochromatic radiation is particularly effective, since the electron beam does not experience brisk changes of the ponderomotive potential during the slippage process.

**Figure 5:**
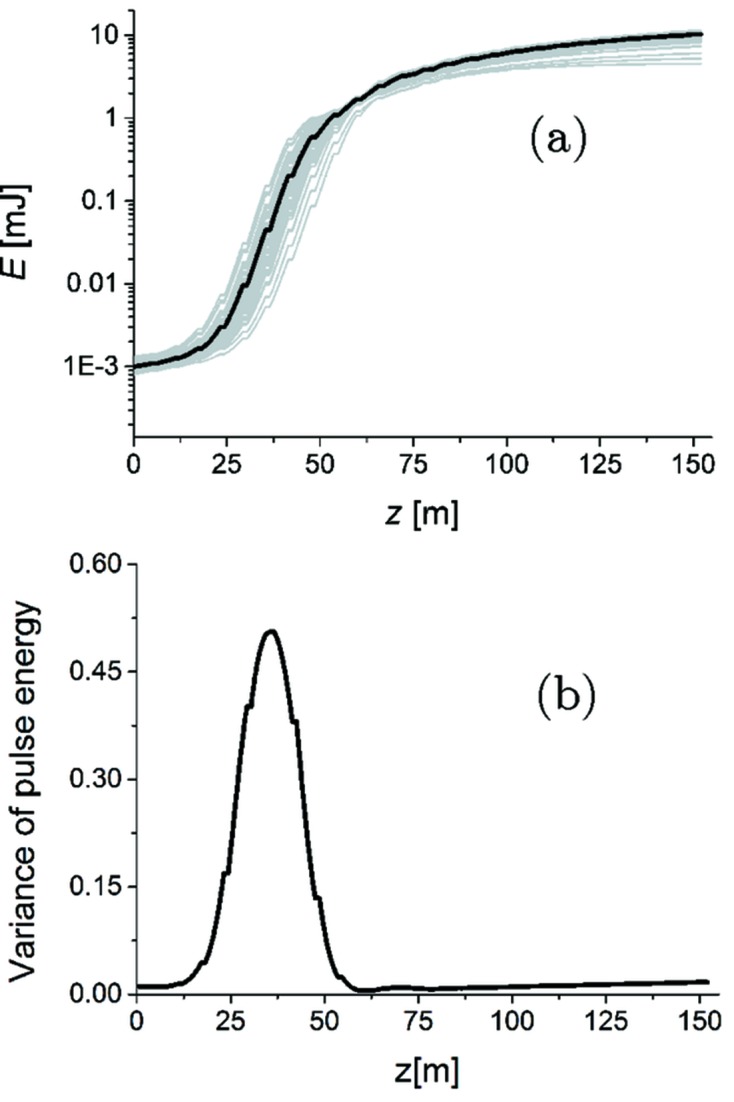
Energy (*a*) and variance (*b*) of energy fluctuations of the seeded FEL pulse as a function of the distance inside the output undulator. Grey lines refer to single-shot realisations, the black line refers to the average over 100 realisations.

**Figure 6:**
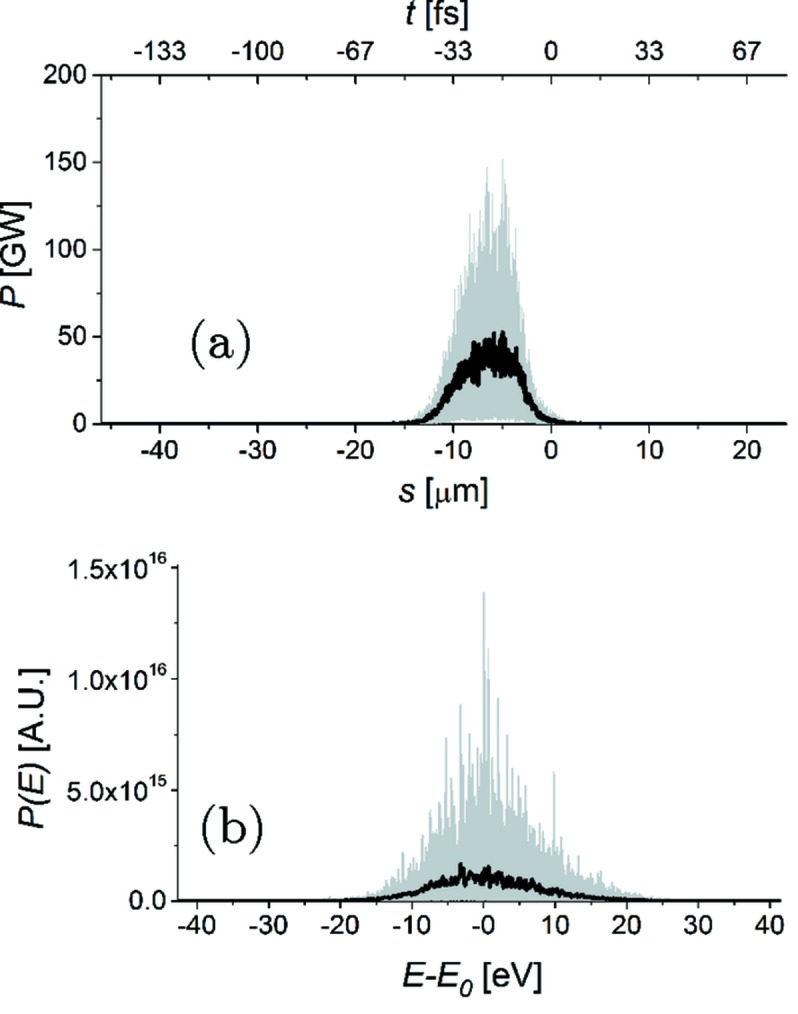
Power (*a*) and spectrum (*b*) in the conventional SASE mode of operation at saturation, to be compared with power and spectrum in the HXRSS mode in Figs. 3(*i*) and 3(*j*) ▸, respectively. Grey lines refer to single-shot realisations, the black line refers to the average over 100 realisations.

The energy and variance of energy fluctuations of the seeded FEL pulse as a function of the distance inside the output undulator are illustrated in Fig. 5 ▸. On the average, pulses of about 11 mJ energy can be produced with this scheme. The final output of our setup is presented in Figs. 3(*i*) and 3(*j*) ▸, in terms of power and spectrum, respectively. This result should be compared with the output power and spectrum for SASE at saturation in Fig. 6 ▸ corresponding to the *conventional* operation mode foreseen at the European XFEL. Considering an average over 100 shots, the peak power for the SASE saturation case in Fig. 6 ▸ is about 4 × 10^10^ W, while for the seeded case in Fig. 3(*i*) ▸ it has grown to 7.5 × 10^11^ W. This corresponds to an increase in flux from about 7 × 10^11^ photons per pulse to about 7 × 10^12^ photons per pulse. This amplification of about one order of magnitude is due to tapering. In addition, the final SASE spectrum has a FWHM of about 11.6 eV, corresponding to a relative bandwidth of 1.2 × 10^−3^ while, due to the enhancement of longitudinal coherence, the seeded spectrum has a FWHM of about 0.94 eV, corresponding to a relative bandwidth of 1 × 10^−4^.

**Figure 7:**
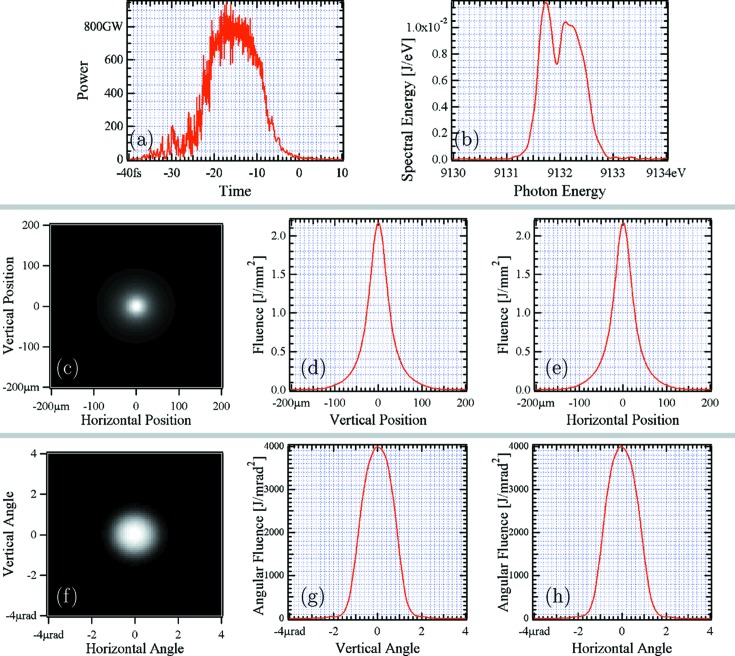
Temporal, spectral, spatial and angular distributions of the radiation pulse at the FEL undulator exit (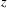 = 74 m in Fig. 8 ▸). (*a*) Pulse power; pulse duration is ∼14 fs (FWHM). (*b*) Spectrum; spectral bandwidth is ∼0.95 eV (FWHM). (*c*) Spatial distribution, two-dimensional plot; (*d*) vertical cut through the center of the fluence distribution; and (*e*) horizontal cut. The beam size is about 50 µm (V) × 50 µm (H) (FWHM). (*f*) Angular distribution, two-dimensional plot; (*g*) vertical cut through the center of the fluence distribution; and (*h*) horizontal cut. The beam divergence amounts to 1.8 µrad (V) × 1.8 µrad (H) (FWHM).

In conclusion, the proposed double-cascade self-seeding tapered scheme yields one order of magnitude increase in peak power due to undulator tapering, and slightly less than an order of magnitude decrease in spectral width due to seeding. Combining the two effects, we obtain an increase in spectral flux density of more that two orders of magnitude compared with saturated SASE (2.1 × 10^14^ photons s^−1^ meV^−1^ compared with 1.5 × 10^12^ photons s^−1^ meV^−1^), in the case where no post-saturation taper is applied. The transverse beam size and divergence at the exit of the undulator are shown in Figs. 7(*c*)–7(*e*) and 7(*f*)–7(*h*) ▸, respectively. The beam profile is nearly circular with a size of about 50 µm (FWHM) and a divergence of about 1.8 µrad (FWHM). In the next section we will complement this information with detailed wavefront propagation simulations through the optical transport line up to the UHRIX setup.

## 3. Optics for ultra-high-resolution IXS   

The desired ultra-high-resolution IXS studies with 0.1 meV spectral and 0.02 nm^−1^ momentum-transfer resolution require a significant amount of X-ray photons with energy 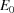 = 9.13185 keV and momentum 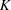 = 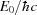 = 46.27598 nm^−1^ to be delivered to the sample within 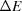 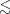 0.1 meV spectral bandwidth and a transverse momentum spread 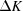 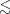 0.02 nm^−1^, all concentrated on the sample in a spot of 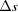 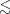 5 µm (FWHM) diameter. The aforementioned photon energy 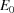 is fixed by the (008) Bragg reflection from Si single crystals, one of the central components of the ultra-high-resolution optics presented in detail below.

**Figure 8:**
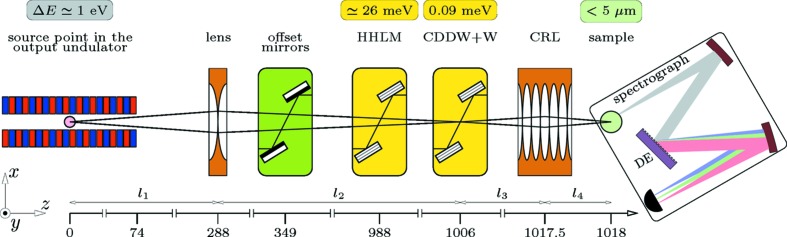
Main optical components of the proposed UHRIX instrument at the SASE-2-undulator beamline of the European XFEL shown schematically together with the output undulator. Optical components are presented as pictographs positioned at various distances from the effective source position in the SASE-2 undulator, 74 m upstream of the undulator exit. See text for descriptions.

We consider a scenario in which the UHRIX instrument is installed at the SASE-2-undulator beamline of the European XFEL. In particular, we consider an option of integrating UHRIX into the Materials Imaging and Dynamics (MID) station (Madsen *et al.*, 2013 ▸), an instrument presently under construction at the European XFEL. A schematic view of the optical components essential for delivering photons with the required properties to the sample is shown in Fig. 8 ▸. Optics are shown as pictographs at certain distances from the source. The effective source position is located around 74 m inside the undulator measured from the exit. This number was determined by back-propagation in free space of the simulated XFEL radiation from the undulator end.

The main optical components are as follows. A biconcave parabolic refractive lens (Lengeler *et al.*, 1999 ▸) creates a secondary source on the six-bounce angular-dispersive ultra-high-resolution CDDW+W monochromator. This is essential in order to achieve a tight focal spot on the sample because it eliminates the blurring that the strong angular dispersion of the CDDW+W monochromator would cause otherwise (Shvyd’ko, 2015 ▸). The CDDW+W monochromator then selects a 0.1 meV spectral bandwidth from the incident X-ray beam. The CDDW+W is a modification of a CDW-type angular-dispersive monochromator (Shvyd’ko *et al.*, 2006 ▸, 2011 ▸; Stoupin *et al.*, 2013 ▸) which uses a three-step process of collimation (C), angular dispersion (D) and wavelength selection (W) (Shvyd’ko, 2004 ▸). Finally, a parabolic compound refractive lens (CRL) (Snigirev *et al.*, 1996 ▸; Lengeler *et al.*, 1999 ▸) focuses the monochromatic X-rays on the sample.

The X-ray spectrograph captures photons scattered from the source in a sufficiently large solid angle and images them in a few-meV wide spectral window with 0.1 meV spectral resolution in the dispersion plane. The dispersing element (DE), a hard X-ray analog of an optical diffraction gratings, is a key component of the spectrograph. The spectrograph is also capable of simultaneously imaging scattered intensity perpendicular to the dispersion plane in the range 0.2 nm^−1^ with 0.01 nm^−1^ resolution. Supplementary optical components include a pair of offset mirrors (*z* = 349 m) which separate the beam from unwanted high-energy bremsstrahlung, and the two-bounce two-crystal non-dispersive high-heat-load monochromator (HHLM at *z* = 988 m). The HHLM narrows the 1 eV bandwidth of the incident X-rays to about 26 meV and thus reduces the heat load onto the CDDW+W monochromator by a factor of 36.

In the remaining parts of this section, the choice of optical elements is justified and their design parameters are determined, first by using dynamical theory calculations for monochromatization with the X-ray crystal optics components in §3.1 and then by applying ray-transfer matrix formalism for ray tracing in §3.3. The optical design is verified by wavefront propagation simulations using a combined application of *GENESIS* (Reiche, 1999 ▸) and *SRW* (Chubar & Elleaume, 1998 ▸) codes with results presented in §3.5.

### 3.1. Monochromatization of X-rays   

The radiation from the undulator discussed previously has about 950 meV bandwidth. It must be reduced to 0.1 meV and delivered to the sample with the smallest possible losses. To this end the previously discussed HHLM and CDDW+W are used in a two-tiered monochromatization scheme. In the following subsections we discuss their operating principles and design parameters in detail.

#### 3.1.1. High-heat-load monochromator   

**Figure 9:**
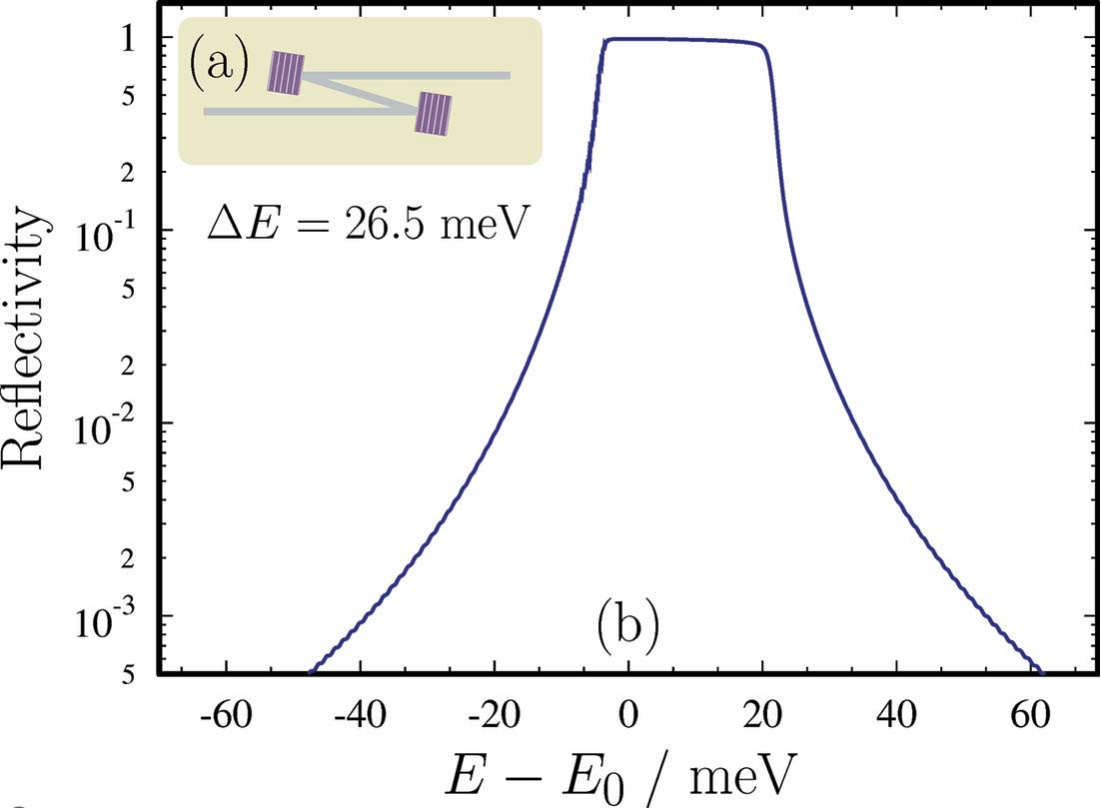
(*a*) Schematic view of the high-heat-load monochromator (HHLM). (*b*) Dynamical theory calculations of the spectral distribution of X-rays around the nominal photon energy 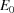 = 9.13185 keV after two successive (115) Bragg reflections from diamond. The spectral bandwidth of the transmitted X-rays is 26.5 meV with a peak reflectivity of 97.7%. The angular spread of the incident X-rays is 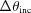 = 1 µrad.

A schematic of the high-heat-load monochromator (HHLM) is shown in Fig. 9(*a*) ▸. In the present design two diamond (C*) crystal plates are used as Bragg reflectors, with the (115) planes parallel to the crystal surface (symmetric Bragg). The (115) reflection is chosen for the Bragg angle to be as close as possible to 90° (backscattering) for 9.13185 keV X-rays. This is dictated by stability requirements under high heat load, as the spectral variation of the reflected X-rays with incidence angle is minimized in back-scattering geometry. The Bragg reflection and crystal parameters used in the HHLM are provided in Table 2 ▸. Dynamical theory calculations of the spectral distribution of X-rays around the nominal photon energy 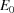 = 9.13185 keV after two successive (115) Bragg reflections from diamond are shown in Fig. 9(*b*) ▸.

#### 3.1.2. High-resolution monochromator CDDW+W   

**Figure 10:**
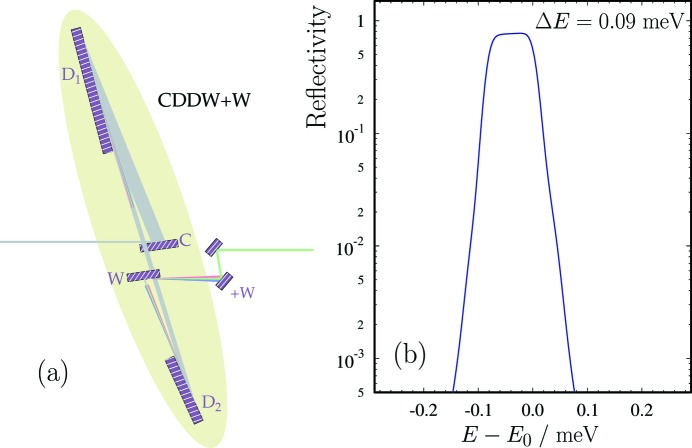
(*a*) Schematic view of the CDDW+W monochromator. (*b*) Dynamical theory calculations of the spectral distribution of X-rays after six successive reflections from the crystals of the CDDW+W optic. Calculations were performed for incident X-rays around the nominal photon energy 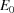 = 9.13185 keV, with an angular spread of 1 µrad and crystal parameters as in Table 3 ▸. The peak reflectivity of the optic is 71% with a spectral bandwidth of 0.09 meV.

The CDDW+W monochromator is a modification of the CDDW monochromator (Shvyd’ko *et al.*, 2011 ▸, 2014 ▸; Stoupin *et al.*, 2013 ▸) complemented by two additional wavelength-selector crystals +W, ensuring a substantially reduced bandwidth and sharp Gaussian tails in the resolution function (Shvyd’ko, 2011 ▸, 2012 ▸; Shvyd’ko *et al.*, 2013 ▸). Fig. 10(*a*) ▸ shows a schematic view of the CDDW+W monochromator, while Fig. 10(*b*) ▸ presents the results of dynamical theory calculations of the spectral distribution of X-rays after the CDDW+W. The crystal parameters used in the calculations are given in Table 3 ▸. The nominal photon energy 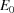 = 9.13185 keV of the UHRIX instrument is determined by the (008) Bragg reflection from the Si dispersion crystals D_1_ and D_2_ with a Bragg angle of 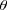 = 89.5°.

### 3.2. Focusing optics   

Because of the very large distances 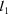 and 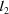 a single two-dimensional parabolic Be lens (Lengeler *et al.*, 1999 ▸), denoted in Fig. 8 ▸ as ‘lens’, is sufficient to focus X-rays onto the CDDW+W monochromator. A lens with 1.68 mm radius (*R*) at the parabola apex, a focal distance 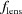 = 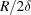 = 205.5 m, and with 1.5 mm geometrical aperture is considered in the following. The corrections 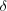 = 4.08684 × 10^−6^ and 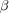 = 1.4201 × 10^−9^ to the refractive index 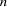 = 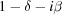 (Henke *et al.*, 1993 ▸) are used in the wavefront-propagation calculations.

The CRL at *z* = 1017.5 m, see Fig. 8 ▸, focuses X-rays from the secondary source at the CDDW+W monochromator onto the sample. In preliminary wavefront propagation simulations an idealized system will be considered consisting of 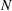 = 39 lenses each of 152.75 µm radius *R* and all placed at the same position. The total focal length of the lens assembly is 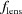 = 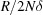 = 0.479 m. In the final calculations a more realistic extended CRL will be used containing 41 individual lenses separated by a 3 mm distance, with the first 39 having a 150 µm radius, and the last two a 400 µm radius at the parabola apex. The geometrical aperture of the CRL is 1 mm, which does not truncate the incident wavefront. All lenses are assumed to be perfect.

### 3.3. Focal spot size and momentum spread on the sample: analytical ray tracing   

**Figure 11:**
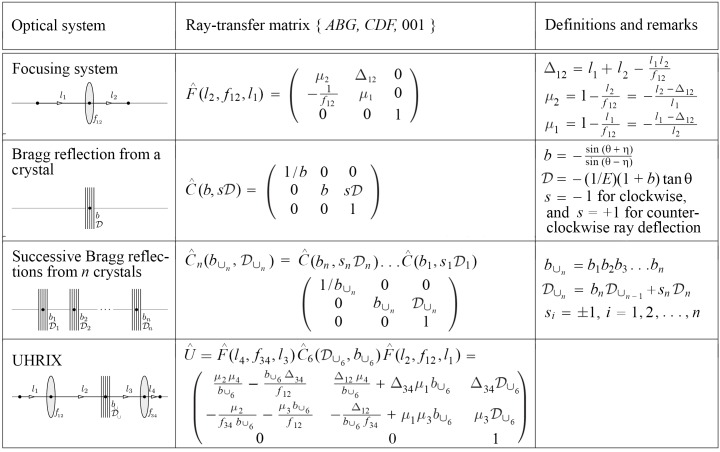
Ray-transfer matrices for a focusing system, for Bragg reflection from crystals, and for the complete optical system of the UHRIX instrument from source to sample.

We use the ray-transfer matrix technique (Kogelnik & Li, 1966 ▸; Matsushita & Kaminaga, 1980 ▸; Siegman, 1986 ▸) to propagate paraxial X-rays through the optical system of the UHRIX instrument and to determine linear and angular sizes of the X-ray beam along the optical system. In a standard treatment, a paraxial ray in any reference plane (a plane perpendicular to the optical axis *z*) is characterized by its distance *x* from the optical axis, by its angle ξ with respect to that axis, and the deviation 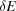 of the photon energy from a nominal value *E*. The ray vector 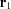
= 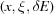 at an input reference plane (source plane) is transformed to 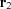 = 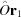 at the output reference plane (image plane), where 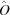 = 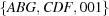 is a ray-transfer matrix of an optical element (elements) placed between the planes. The upper three rows of Fig. 11 ▸ present the ray-transfer matrices of the major components of the UHRIX optical system. The ray-transfer matrix 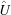 of the UHRIX instrument, which describes propagation from the source to the sample, is presented in the last row of Fig. 11 ▸. We refer to Shvyd’ko (2015 ▸) for details about the derivation of these matrices and provide here only essential notation and definitions.

In the focusing system, see the matrix 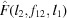 in Fig. 11 ▸, a source in a reference plane at a distance 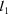 upstream of a lens with focal length 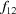 is imaged onto the reference image plane located at a distance 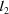 downstream from the lens. If the parameter 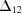 defined in Fig. 11 ▸ equals zero, the classical lens equation 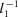 + 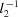 = 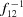 holds. In this case the system images the source with inversion and a magnification factor 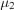 = 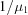 = 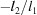 independent of the angular spread of rays in the source plane.

In the ray-transfer matrix 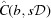, describing Bragg reflection from a crystal at angle θ, the asymmetry factor *b* determines how the beam size and divergence change upon Bragg reflection. The angular dispersion rate 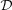 describes how the photon energy variation 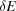 from a nominal value *E* changes the reflection angle with a fixed incident angle. The Bragg reflecting atomic planes are assumed to be at an asymmetry angle η with respect to the crystal surface.

The ray-transfer matrix 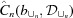, describing successive Bragg reflections from a system of *n* crystals, has the same structure as that of a single Bragg reflection. The only difference is that the asymmetry parameter *b* and the angular dispersion rate 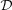 are substituted by the appropriate cumulative values 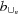 and 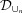, respectively. The ray-transfer matrices of the offset mirrors and of the HHLM consisting of two symmetric Bragg reflections (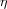 = 0, 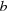 = −1, 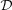 = 0) (see Table 2 ▸) are unit matrices, leading to no change in the beam parameters.

The total ray transfer matrix 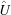 of the UHRIX instrument is a product of the ray-transfer matrices of the lens focusing system 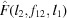, the CDDW+W six-crystal matrix 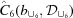 and of the CRL focusing system 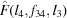. The asymmetry parameters and the dispersion rate of the CDDW+W monochromator crystals required for the CDDW+W matrix are provided in Table 3 ▸. 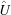 describes propagation of X-rays in the vertical 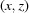 plane (see reference system in Fig. 8 ▸), in which the Bragg diffraction from the monochromator crystals takes place. Propagation of X-rays in the horizontal 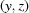 plane is not affected by Bragg diffraction from the monochromator crystals. Here, the appropriate UHRIX ray-transfer matrix is obtained from 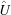 with parameters 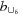 = 1 and 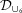 = 0.

To determine the actual focal size and angular spread on the sample we use a linear source size (FWHM) 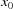 = 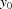 = 50 µm, and an angular source size 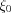 = 1.8 µrad, as derived from the XFEL simulations in §2. The energy spread of the X-rays is assumed to be 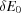 = 0.09 meV. For the cumulative asymmetry parameter and dispersion rate of the CDDW+W monochromator we use 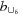 = 2.25 and 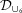 = 112 µrad meV^−1^ as obtained from Table 3 ▸ and the distances between the optical elements are 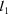 = 288 m, 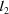 = 718 m, 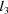 = 11.5 m and 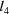 = 0.5 m (see Fig. 8 ▸).

#### 3.3.1. Focal spot size on the sample   

The smallest focal spot size on the sample is achieved provided 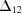 = 0, that is, the lens focuses X-rays on the CDDW+W monochromator, and 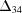 = 0, meaning that the CRL refocuses X-rays on the sample with the secondary source on the CDDW+W monochromator. The focusing conditions require 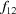 = 205.5 m and 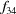 = 0.479 m for the focal distances for the lens and CRL, respectively (see also §3.2). In this case the elements *B* and *G* of the 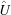 matrix are zero so the vertical and horizontal linear sizes of the source image on the sample are determined only by the element *A*:
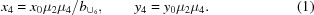
With 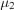 = 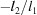 = 2.5 and 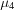 = 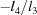 = 0.044, we obtain for the vertical spot size 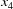 = 2.4 µm, while for the horizontal size 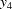 = 5.4 µm. The vertical spot size 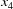 is less than half the target specification (5 µm) required to achieve 0.1 meV spectral resolution of the spectrograph (Shvyd’ko, 2015 ▸), as discussed below in §3.4. If focusing onto the CDDW+W is not perfect so that 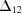 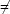 0 , this may lead to an increase in the spot size by 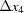 = 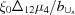 (resulting from element *B* of the UHRIX ray-transfer matrix). However, this is not very critical as, even with a mismatch of 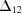 ≃ 10 m, the spot size increases only by an insignificant 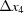 ≃ 0.4 µm.

#### 3.3.2. Transverse momentum spread   

The transverse momentum spread in the diffraction plane (vertical) 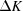 = 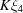 is defined by the angular spread
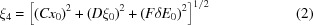
of X-rays incident on the sample.^5^ Here we assume a Gaussian distribution of the beam parameters. In the vertical scattering plane the UHRIX ray-transfer matrix elements are 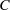 = 2.56 µrad µm^−1^, 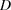 = 21 and 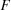 = −2.58 µrad µeV^−1^. With 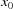 = 50 µm, 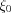 = 1.8 µrad and 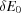 = 90 µeV we obtain 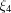 = 265 µrad and 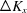 = 0.012 nm^−1^.

In the horizontal plane there is no angular dispersion. The cumulative dispersion rate 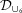 = 0 and the asymmetry parameter 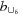 = 1. As a result, the angular dispersion related term 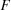 = 0 and the only two non-zero elements are 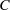 = 5.31 µrad µm^−1^ and 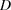 = 9, resulting in 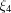 = 266 µrad and 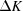 = 0.012 nm^−1^. We note that both the vertical and the horizontal momentum spreads are smaller than the target specification 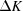 = 0.02 nm^−1^.

#### 3.3.3. Pulse dilation   

Bragg diffraction from an asymmetrically cut crystal with angular dispersion rate 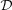 inclines the X-ray intensity front by an angle 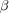 = 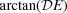 resulting in a pulse dilation 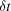 = 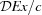 (Shvyd’ko & Lindberg, 2012 ▸) along the optical axis *z*. Here *x* is the transverse pulse size after the angular dispersive optics and *c* is the speed of light in a vacuum. This effect is similar to wavefront inclination by optical diffraction gratings. The multi-crystal CDDW+W optic has a very large cumulative angular dispersion rate 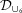 = 112 µrad meV^−1^ (see Table 3 ▸). The result is an inclination of the pulse intensity front by 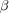 = 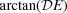 = 89.94° and thus a very large pulse stretching 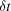 = 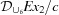 = 190 ps (equivalent to a 57 mm pulse length). Here, 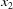 = 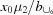 = 56 µm is the vertical beam size after the CDDW monochromator.

### 3.4. Spectrograph   

Spectral analysis of photons scattered from the sample is another important component of IXS spectrometers. Unlike monochromators, spectral analyzers should have a large angular acceptance, capable of collecting photons from the greatest possible solid angle (limited only by the required momentum transfer resolution), and with a spectral resolution matched to that of the monochromator. The spectral analyzer is usually the most difficult part of IXS spectrometers. In a standard approach the IXS analyzers measure sequentially one spectral point after another. A better strategy is to image the entire or a large part of the IXS spectra in single shots. Therefore, in the IXS instrument proposed here, the photon spectra are measured by an X-ray spectrograph. A spectrograph is an optical instrument that disperses photons of different energies into distinct directions and space locations, and images photon spectra on a position-sensitive detector. Spectrographs consist of collimating, angular-dispersive and focusing optical elements. Their principal schematic is shown in the pictograph of Fig. 8 ▸. Bragg reflecting crystals arranged in an asymmetric scattering geometry are used as dispersing elements (DE) of the hard X-ray spectrograph studied here (Shvyd’ko, 2011 ▸, 2012 ▸, 2015 ▸; Shvyd’ko *et al.*, 2013 ▸).

**Figure 12:**
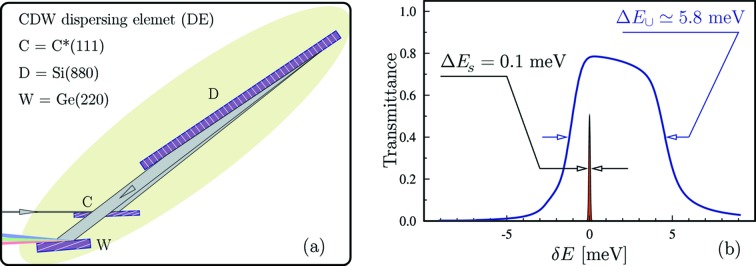
(*a*) CDW-type three-crystal dispersing element of the spectrograph. (*b*) Spectral transmission function of the spectrograph with the CDW dispersing element ensuring a 5.8 meV broad window of imaging. The sharp line presents a 0.1 meV design spectral resolution of the spectrograph.

Several optical designs of hard X-ray spectrographs were proposed and their performances analyzed by Shvyd’ko (2015 ▸). Spectrographs with the desired target energy resolution of 0.1 meV and a spectral window of imaging up to a few tens of meV were shown to be feasible for IXS applications. We refer to Shvyd’ko (2015 ▸) for details. Here, we only briefly outline a particular spectrograph design with a DE consisting of three crystals in a CDW arrangement, schematically shown in Fig. 12(*a*) ▸. Fig. 12(*b*) ▸ shows the spectrograph’s spectral transmission function with a 5.8 meV-wide window of imaging. The sharp line in the same figure represents the 0.1 meV design resolution.

The spectral resolution of the spectrograph is given by
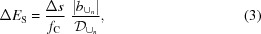
derived using the ray-transfer matrix formalism [see §3.3 and Shvyd’ko (2015 ▸)]. A large cumulative dispersion rate 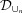 of the dispersing element, a small cumulative asymmetry factor 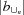, a large focal distance 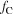 of the collimating optics, and a small source size 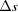 (beam size on the sample) are advantageous for better spectral resolution. For the three-crystal CDW dispersing element, with the optical scheme depicted in Fig. 12(*a*) ▸, we have 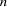 = 3, 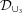 = 25 µrad meV^−1^ and 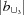 = 0.5. The target resolution of 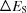 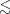 0.1 meV is attained with 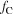 = 1 m and 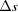 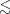 5 µm. The latter is in fact the origin of the target specification for the focal spot size on the sample discussed in the beginning of §3. The estimated design value 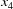 = 2.4 µm, see §3.3.1, is half the specification value and hence should yield a two times better spectral resolution than the 0.1 meV at target.^6^ For spectral imaging, focusing onto the detector is required only in one dimension. Hence, with a two-dimensional position-sensitive detector it is possible to simultaneously image the spectrum of X-rays along the vertical axis and the momentum transfer distribution along the horizontal axis.

### 3.5. Wavefront propagation through UHRIX optics   

In this section the design parameters of the UHRIX are verified by wavefront propagation calculations. Physical optics simulations of the interaction of X-rays with the various optical elements of Fig. 8 ▸ have been performed with the aid of two programs. The first, *GENESIS* (Reiche, 1999 ▸), calculates the original wavefront of the SASE radiation at the exit of the output undulator, with the results presented in §2.2. The second, *SRW* (Chubar & Elleaume, 1998 ▸), calculates the wavefront after propagation from the undulator through drift spaces and optical components by using Fourier-optics-compatible local propagators. Altogether, including all lenses, crystals and drift spaces, the beamline contains more than 100 elements. Simulations of the diffracting crystals with *SRW* have only recently become possible by addition of a new module (Sutter *et al.*, 2014 ▸) which also has been applied to the design of the planned IXS beamline at NSLS-II (Suvorov *et al.*, 2014 ▸).

The temporal, spectral, spatial and angular radiation pulse distributions and their parameters at the FEL undulator exit, 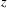 = 74 m in Fig. 8 ▸, are given in Fig. 7 ▸. Radiation parameters (FWHM) such as pulse duration 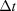, spectral width 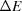, transverse size 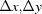, angular spread 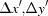, and transverse momentum spread 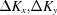 are provided in the caption of Fig. 7 ▸ and summarized in Table 4 ▸ together with peak and average flux values. The peak values are also a result of averaging over 100 runs with *GENESIS*, as discussed in §2.2. The average flux values are obtained assuming a pulse repetition rate of 27 kHz.

**Figure 13:**
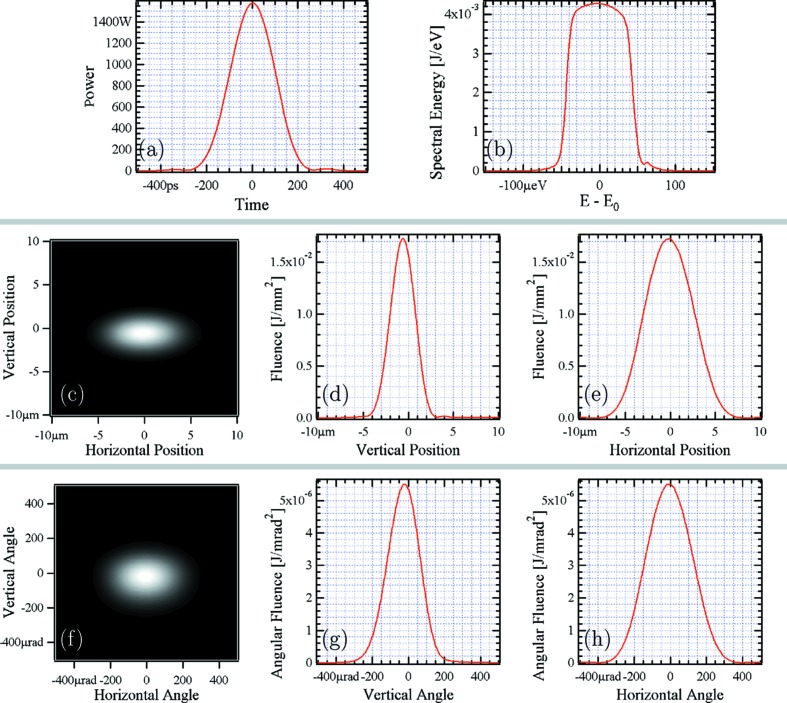
Temporal, spectral, spatial and angular distributions of the radiation pulse on the sample (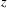 = 1018 m in Fig. 8 ▸). (*a*) Pulse power; the pulse duration is ∼225 ps (FWHM). (*b*) Spectrum; the spectral bandwidth is ∼0.090 meV (FWHM). (*c*) Two-dimensional plot of the spatial distribution. (*d*) Vertical cut through the maximum of the fluence distribution; and (*e*) horizontal cut. The beam size on the sample is 3.3 µm (V) × 6.5 µm (H) (FWHM). (*f*) Angular distribution, two-dimensional plot; (*g*) vertical cut through the maximum of the fluence distribution; and (*h*) horizontal cut. Beam divergence on the sample is 220 µrad (V) × 310 µrad (H) (FWHM), corresponding to a 0.01 nm^−1^ × 0.015 nm^−1^ transverse momentum spread.

Results of the wavefront propagation simulations related to the sample area are presented graphically in Fig. 13 ▸. The temporal, spectral, spatial and angular radiation pulse distributions and their parameters at the sample location (image plane), 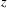 = 1018 m in Fig. 8 ▸, are provided in the captions of Fig. 13 ▸ and summarized in Table 4 ▸ together with the peak and average flux values on the sample. The calculated radiation parameters at the sample location are in good agreement with values obtained by the ray-transfer matrix approach (§3.3) which are shown for comparison in Table 4 ▸. They are also in agreement with the target specifications for the UHRIX instrument defined in §3.

#### 3.5.1. Spectral, spatial and angular distribution   

**Figure 14:**
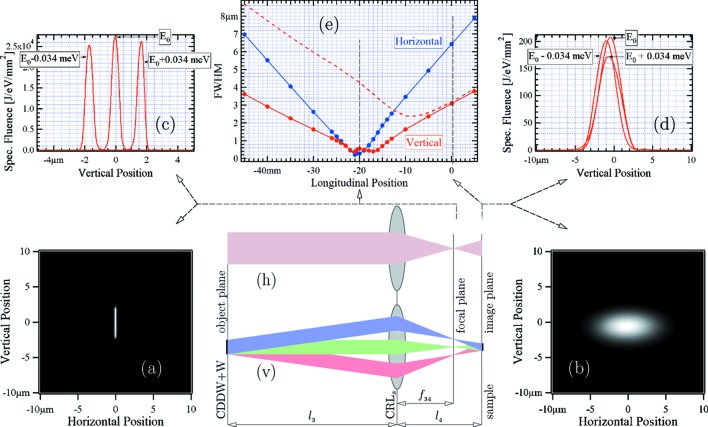
Fluence distributions and spot sizes of X-rays at different longitudinal positions near the sample. (*a*) Fluence distribution near the focal plane, and (*b*) in the sample (image) plane integrated over photon energies or pulse duration. (*c*) Vertical cuts through spectral fluence distributions at zero horizontal position for different spectral components near the focal plane (−20 mm), and (*d*) in the image plane (0 mm). (*e*) Vertical and horizontal spot sizes (FWHM) for the monochromatic radiation component 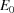 as a function of longitudinal position along the beam are presented by the solid lines. The red dashed curve in (*e*) represents the vertical size integrated over all spectral components. The optical scheme and schematic of ray propagation in the CRL focusing system are presented both in the vertical (v) and horizontal planes (h). The CDDW+W monochromator is in the object plane while the sample is in the image plane.

To avoid enlargement of the beam size on the sample due to the angular dispersion in the CDDW+W monochromator, it was proposed to place this monochromator in the object plane of the CRL (see §3.3.1). This works perfectly in the geometrical optics approximation if the monochromator and the CRL are assumed to be point-like [see §3.3.1, and also the schematics (v) and (h) in Fig. 14 ▸]. The question is how well this works with realistic sizes of monochromator crystals and of the individual lenses in the CRL, and with non-zero distances between all these elements. To address these issues, wavefront propagation simulations have been performed under realistic conditions. Detailed results are presented in Fig. 14 ▸, showing fluence distributions and spot sizes of X-rays at different longitudinal positions near the sample. There are striking differences in the transverse shape and sizes, integrated over all spectral components, in the image plane (Fig. 14*b* ▸) and in the focal plane (Fig. 14*a* ▸). There are equally striking differences in the positions and widths of the vertical beam profiles for different spectral components in the image plane (Fig. 14*d* ▸) and in the focal plane (Fig. 14*c* ▸).

The widths of the vertical pulse profiles (FWHM) for the monochromatic component 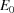 at different locations are presented in Fig. 14(*e*) ▸ by the red solid line. The blue solid line shows the widths of the horizontal profiles. The smallest widths, 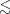
0.5 µm, of the vertical and horizontal monochromatic pulse profiles are achieved at ∼21 mm upstream of the sample position. This location coincides with the location of the focal plane, which is at a distance of 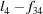 = 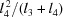 = 21 mm from the CRL center [see sketches (v) and (h) in Fig. 14 ▸]. In the image plane the vertical width of approximately 3 µm is much larger but all monochromatic profiles are almost at the same position so they probe the same scattering volume, as shown in Fig. 14(*d*) ▸. This is in agreement with the ray-transfer matrix calculations predicting zero linear dispersion in the image plane, as desired. In contrast, in the focal plane different monochromatic components are focused to much smaller sizes (∼0.5 µm) but without spatial overlap, as shown in Fig. 14(*c*) ▸.

Sketch (v) in Fig. 14 ▸ illustrates the origin of this behavior: each monochromatic radiation component emanates from the CDDW+W monochromator (located in the first approximation in the object plane) with a very small angular spread 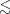
2 µrad. Therefore, with a virtual source position practically at infinity, they are focused onto the focal plane. Different monochromatic components emanate at different angles because of strong angular dispersion in the CDDW+W monochromator that eventually results in a linear dispersion in the vertical direction of the focal plane but no dispersion in the image plane, as required for UHRIX.

The horizontal transverse size of the X-ray pulse is independent of photon energy, since angular dispersion in the CDDW+W monochromator takes place only in the vertical plane. The smallest horizontal beam size is achieved near the focal plane with ∼0.3 µm^7^ [see Figs. 14(*a*) and Fig. 14(*c*) ▸]. This occurs because of the very small horizontal angular spread, 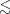
1 µrad, of all X-ray spectral components emanating from the CDDW+W monochromator.

We note that the best position for the sample is actually neither in the image plane nor in the focal plane. As follows from the dependence presented by the dashed line in Fig. 14(*e*) ▸, the smallest vertical beam size averaged over all spectral components is ∼2.5 µm and it is achieved at about −10 mm from the image plane. The horizontal beam size at the same position is ∼3.5 µm. We also note that the extended (realistic three-dimensional model) CRL described in §3.2 does not introduce any substantial differences with respect to the initial simulations with an idealized thin CRL.

#### 3.5.2. Spatiotemporal distributions   

The strong angular dispersion in the CDDW+W monochromator also causes substantial pulse dilation, as ray-transfer matrix calculations have shown in §3.3.3. Here we present and discuss results of calculations of the spatiotemporal distributions of the X-ray pulses obtained by the wavefront propagation simulations.

The pulse duration at the exit of the undulator is only 15 fs (FWHM), as shown in Fig. 7 ▸. The pulse spectral bandwidth is ∼950 meV and it is reduced to 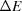 = 0.09 meV (FWHM) by the crystal monochromators. Assuming a Gaussian spectral distribution after the CDDW+W monochromator, we obtain for the duration of a Fourier-transform-limited pulse 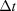 = 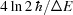 = 18.2 ps (FWHM). The results of the calculations shown in Fig. 13 ▸ predict, however, a more than an order of magnitude larger pulse duration of ∼225 ps. This number agrees well with the duration calculated in §3.3.3 as a result of the wavefront inclination caused by angular dispersion in the CDDW+W monochromator.

#### 3.5.3. Wavefront propagation summary   

The wavefront propagation simulations confirm the soundness of the optical design of the UHRIX instrument worked out initially by the ray-transfer matrix approach and dynamical theory calculations. They also confirm the feasibility of the target specifications. The simulations show that the spectral flux from the XFEL undulator can be transported to the sample through the UHRIX X-ray optics with 30% efficiency reaching a remarkably high value of ∼7 × 10^13^ photons s^−1^ meV^−1^. This number exceeds by more than three orders of magnitude the spectral flux numbers reported for state-of-the-art IXS instruments at synchrotron radiation facilities (Baron, 2015 ▸). Custom-designed crystal and focusing optics ensure that on the sample ∼6.3 × 10^12^ photons s^−1^ meV^−1^ photons can be concentrated in a spectral band of 0.09 meV in a spot of 3.3 µm (V) × 6.5 µm (H) size and with a momentum transfer spread of 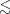
0.015 nm^−1^.

## 4. Discussion and conclusions   

This article explores novel opportunities for ultra-high-resolution IXS (UHRIX) at high-repetition-rate XFELs unlocked by the recent demonstration of a conceptually new spectrometer (Shvyd’ko *et al.*, 2014 ▸) with unprecedented specifications (0.6 meV spectral resolution and 0.25 nm^−1^ momentum transfer), operating around 9 keV. Its exploitation, together with the broadband ultra-high-resolution imaging spectrograph proposed by Shvyd’ko (2015 ▸), will make it possible to fill the energy–momentum gap between high- and low-frequency inelastic probes and to provide exciting new opportunities for studies of dynamics in condensed matter. In particular, UHRIX experiments can be enabled at the European XFEL, where an increase of more than three orders of magnitude in average spectral flux is expected compared with what is available today at synchrotrons. The gain is due to two main factors: firstly, the high repetition rate of the European XFEL, owing to the superconducting linac accelerator driver, which allows up to 27000 X-ray pulses per second, and, secondly, the presence of long undulators, allowing the combined implementation of hard X-ray self-seeding (HXRSS) and post-saturation tapering techniques. In particular, a double-chicane HXRSS scheme increases the signal-to-noise ratio and eases the heat load on the HXRSS crystals to a tolerable level. This scheme is expected to yield up to TW-level X-ray pulses. Simulations of pulse propagation up to the sample position through the UHRIX optics show that an unprecedented average spectral flux of 7 × 10^13^ photons s^−1^ meV^−1^ is feasible. The power delivered to the sample can be as high as 350 W mm^−2^ and radiation damage can become a limitation but liquid jets and scanning setups for solid samples can be employed to circumvent eventual problems (see Madsen *et al.*, 2013 ▸, and references therein).

## Figures and Tables

**Table 1:** Operation parameters of the European XFEL used in this paper (*) refers to the position in the bunch with maximum peak current.

		Units
Undulator period	40	mm
Periods per segment	125	
Total number of segments	35	
*K* parameter (r.m.s.)	2.658	
Intersection length	1.1	m
Wavelength	0.1358	nm
Energy	17.5	GeV
Charge	250	pC
Horizontal normalized slice emittance (*)	4.0 × 10^−7^	m rad
Vertical normalized slice emittance (*)	3.6 × 10^−7^	m rad
Peak current	5.0	kA
Energy spread σ_γ_ (*)	0.96	

**Table 2:** Crystal and Bragg reflection parameters of the crystal elements of the HHL monochromator 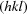
: Miller indices of the Bragg diffraction vector **H**. 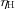: asymmetry angle. 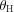: glancing angle of incidence. *d*: crystal thickness. 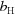 = 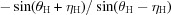: asymmetry parameter. 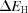 and 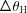 are the Bragg reflection’s intrinsic spectral width and angular acceptance, respectively.

Crystal / function	**H** (*hkl*)	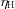 (°)	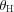 (°)	*d* (mm)	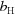	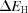 (meV)	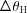 (µrad)
C* / 1st	(1 1 5)	0	81.45	0.1	−1	33	24
C* / 2nd	(1 1 5)	0	81.45	0.3	−1	33	24

**Table 3:** Elements of the CDDW+W optics with their crystal and Bragg reflection parameters Similar definitions are used as in Table 2 ▸. In addition, 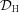 is the Bragg reflection’s dispersion rate. The cumulative asymmetry parameter and dispersion rate of the monochromator are 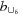 = 2.25 and 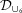 = 112 µrad meV^−1^; see definition in Figure 11 ▸. The X-ray photon energy is 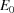 = 9.13185 keV.

Crystal / function	**H** (*hkl*)	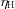 (°)	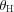 (°)	*d* (mm)	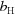	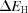 (meV)	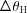 (µrad)	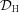 (µrad meV^−1^)
C* / C	(3 3 1)	−48	56.06	0.5	−0.14	124	20	−0.1
Si / D_1_	(8 0 0)	87.5	89.5	10	−1.5	22	280	6.2
Si / D_2_	(8 0 0)	87.5	89.5	10	−1.5	22	280	−6.2
C* / W	(3 3 1)	48	56.05	0.5	−6.9	18	2.9	0.9
C* / +W	(4 0 0)	0	49.57	0.5	−1.0	75	10	0
C* / +W	(4 0 0)	0	49.57	0.5	−1.0	75	10	0

**Table 4:** Values (FWHM) of X-ray pulse parameters at different locations along the beamline in HXRSS mode with the UHRIX setup See text for details. The total transmittance of the optics is 30%.

Location (method)	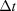 (ps)	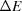 (meV)	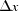 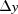 (µm)	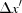 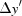 (µrad)	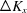 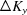 (nm^−1^)	Pulse energy (µJ)	Photons/pulse (photons pulse^−1^)	Flux (photons s^−1^)	Spectral flux (photons s^−1^ meV^−1^)
Undulator exit, *z* = 74 m (*GENESIS*)	0.014	950	50	1.8	0	11000	7.5 × 10^12^	2.0 × 10^17^	2.1 × 10^14^
			50	1.8	0				

Sample, *z* = 1018 m (*SRW* wavefront propagation)	225	0.087	3.3	220	0.01	0.33	2.3 × 10^8^	6.3 × 10^12^	7 × 10^13^
			6.5	310	0.015				

Sample, *z* = 1018 m (ray-transfer matrix)	190	0.09	2.4	265	0.012				
			5.5	266	0.012				

## Footnotes

1 INS cannot enter this region due to the kinematic limitation. The low-frequency probes cannot enter this region because their photon wavelengths are too long.

2 A Fourier-transform IXS technique has been demonstrated recently (Trigo *et al.*, 2013 ▸), which can be considered as a powerful complementary approach for studies of *non-equilibrium* excitations with ultra-high spectral resolution.

3 After successful demonstration of the self-seeding setup with a single-crystal monochromator at the LCLS, it was decided that a double-cascade self-seeding scheme should be enabled at the SASE-2 beamline of the European XFEL from an early stage of operation (XFELSEED, 2014 ▸).

4 More precisely, that study considered X-ray pulses of 3 µJ, with a transverse size of 35 µm FWHM, an energy of 8.2 keV at a repetition rate of 4.5 MHz. In that case the drift of the central frequency for 1000 pulses is within the Darwin width of reflection.

5 The beam sizes and the angular spread in equations (1) and (2) are obtained by propagation of second-order statistical moments, using transport matrices derived from the matrices presented in Fig. 11 ▸, and assuming zero cross-correlations (*i.e.* zero mixed second-order moments).

6 We note that with increasing scattering angle the sample thickness starts to play a role, contributing to a ‘projected’ scattering source size.

7 The small horizontal beam size near the focal plane could be used to substantially improve the resolution of the spectrograph [see equation (3)]. For this, however, its dispersion plane has to be oriented horizontally, and the sample placed into the focal plane. Alternatively, the dispersion plane of the CDDW+W monochromator could be oriented horizontally, to produce a very small vertical beam size on the sample in the focal plane.
